# Glycol chitosan nanoparticles as specialized cancer therapeutic vehicles: Sequential delivery of doxorubicin and Bcl-2 siRNA

**DOI:** 10.1038/srep06878

**Published:** 2014-11-03

**Authors:** Hong Yeol Yoon, Sejin Son, So Jin Lee, Dong Gil You, Ji Young Yhee, Jae Hyung Park, Maggie Swierczewska, Seulki Lee, Ick Chan Kwon, Sun Hwa Kim, Kwangmeyung Kim, Martin G. Pomper

**Affiliations:** 1 Center for Theragnosis, Biomedical Research Institute, Korea Institute of Science and Technology (KIST), Seoul 136-791, South Korea; 2 School of Chemical Engineering, Sungkyunkwan University, Suwon 440-746; 3 The Russell H. Morgan Department of Radiology and Radiological Science, Johns Hopkins School of Medicine, Baltimore, Maryland 21287, USA; 4 Center for Nanomedicine at the Wilmer Eye Institute, Johns Hopkins School of Medicine, Baltimore, Maryland 21287, USA; 5 KU-KIST School, Korea University, Seoul 136-701, South Korea

## Abstract

Conventional chemotherapy is plagued with adverse side effects because cancer treatments are subject to numerous variations, most predominantly from drug resistance. Accordingly, multiple or multistage chemotherapeutic regimens are often performed, combining two or more drugs with orthogonal and possibly synergistic mechanisms. In this respect, glycol chitosan (GC)-based nanoparticles (CNPs) serve as an effective platform vehicle that can encapsulate both chemotherapeutics and siRNA to achieve maximal efficacy by overcoming resistance. Herein, DOX-encapsulated CNPs (DOX-CNPs) or Bcl-2 siRNA-encapsulated CNPs (siRNA-CNPs) exhibited similar physicochemical properties, including size, surface properties and pH sensitive behavior, regardless of the different physical features of DOX and Bcl-2 siRNA. We confirmed that the CNP platform applied to two different types of drugs results in similar *in vivo* biodistribution and pharmacokinetics, enhancing treatment in a dose-dependent fashion.

Despite the importance of chemotherapy for treating cancer, many such agents eventually become impotent in the face of tumor heterogeneity and the development of multi-drug resistance. Cancer treatments are subject to more variation than treatments for most other diseases because of drug resistance and the complex tumor microenvironment, especially concerning cancers with low survival rates^1^,^2^,^3^. Accordingly, various treatment strategies have been attempted to overcome drug resistance and heterogeneity depending on the stage and type of tumor^4^.

Recently, multiple or multi-stage chemotherapeutic regimens have been adopted by clinicians to overcome the above mentioned challenges to treatment. Because first-line therapies often fail, patients undergo progression or recurrence requiring second and third line combination chemotherapy^5^,^6^,^7^. The treatment of two or more drugs simultaneously can be more effective. Through a suitable combination of drugs, comprised of gene and/or chemotherapeutics, efficacy could be maximized and drug resistance could be overcome^8^.

Most conventional chemotherapeutic and genetic anti-cancer agents, the latter including DNA, siRNA and miRNA, have poor pharmacokinetic profiles, and both drug classes are distributed non-specifically in the body leading to systemic toxicity associated with serious side effects^9^,^10^,^11^. Above all, the effective combination of chemotherapeutics and genes is particularly challenging since both agents exhibit quite different physicochemical properties, inevitably resulting in different pharmacokinetics and behaviors *in vivo*. In this context, safe and highly qualified vehicles for combinational delivery carrying different types of drugs are required to satisfy the recent clinical demand for multiple and multi-stage therapy. In spite of the constant effort towards developing efficient nano-sized drug carriers^12^,^13^,^14^,^15^, little attention has been paid to developing nano-sized drug carriers appropriate for simultaneous delivery of chemotherapeutics and genes to enhance the efficacy of combinatorial cancer treatment.

Herein, tumor-homing and biocompatible glycol chitosan (GC)-based nanoparticles (CNPs) can serve as a platform vehicle to encapsulate both chemotherapeutics and siRNA to achieve optimal efficacy. Also, CNPs for sequential delivery of DOX and Bcl-2 siRNA may overcome the problem of drug resistance. Among several combinations of chemotherapeutics, Bcl-2 specific siRNA (siBcl2) has been considered as an attractive choice, since most of the anticancer therapeutics including DOX, paclitaxel and docetaxel trigger apoptosis mechanism and simultaneously activate cellular defense mechanism of anti-apoptosis, which prevents cell death^16^,^17^. DOX and Bcl-2 siRNA were simply encapsulated into CNPs by hydrophobic and charge-charge interactions, respectively, resulting in DOX encapsulated CNPs (DOX-CNPs) and Bcl-2 siRNA encapsulated CNPs (siRNA-CNPs). Importantly, our CNP-based delivery platform exhibited nearly similar physico-chemical properties in size, surface properties and pH sensitive behavior, despite the widely disparate physical features of DOX and siRNA. That aspect resulted in efficient tumor targeting CNPs^18^,^19^,^20^,^21^,^22^, which may serve as a suitable combinatorial anti-cancer regimen. The remarkably long-term and enhanced anti-cancer efficacy, without noticeable systemic toxicity, can be explained by the tendency for the CNPs to accumulate within tumor, followed by extravasation of their contents from the tumor vessels to the surrounding tissues. Moreover, we demonstrate that the mode and timing of administration of the CNPs are key elements required to achieve optimal efficacy.

## Results and Discussion

**Figure 1:**
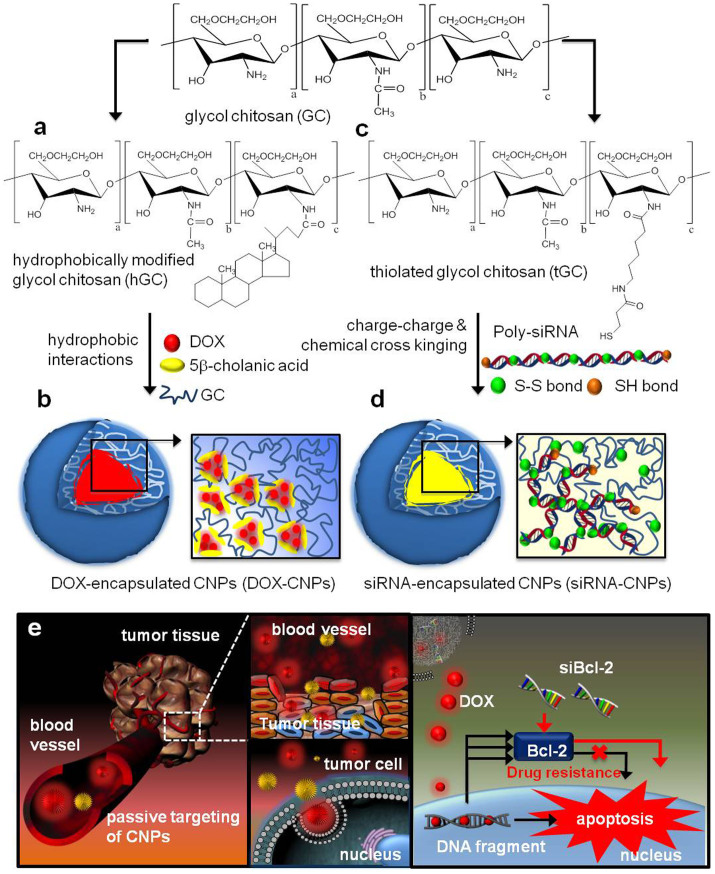
(a) Synthetic scheme of hydrophobic 5β-cholanic acid modified GC. (b) Schematic illustration of effective self-assembly of DOX-CNPs by hydrophobic interactions between DOX and cholanic acid moieties of GC polymers in mild conditions. (c) Synthetic scheme of thiolated glycol chitosan (tGC) by chemical conjugation of Sulfo-LC-SPDP to the amine group of GC polymers. (d) Schematic illustration of fthe ormulation process of small and compact Poly-siRNA/tGC complexes. First, weak charge interactions occur between negatively charged Poly-siRNA and positively charged GC polymers. Next, Poly-siRNA/tGC complexes are further stabilized by chemical disulfide crosslinking between tGC and Poly-siRNA. (e) Schematic representation of the combinational delivery process by GC-based nano-platforms. CNP-based delivery platforms exhibit almost similiar physico-chemical properties after encapsulation of drugs with extremely different physical features, i.e. DOX and siBcl2. Finally DOX-CNPs and siRNA-CNPs show outstanding tumor accumulation and localization tendencies thereafter similar cellular uptake followed by intracellular trafficking to the site of action, cytosol for the RNAi process and nucleus for DOX action, which will benefit anti-cancer therapeutic effects in a combinatorial way.

A chemically modified GC polymer (MW = 250 kDa) was utilized as the chemotherapeutics and siRNA delivery vehicles. First, hydrophobically modified GCs were synthesized by chemical conjugation of hydrophobic 5β-cholanic acid (CA, 158 ± 6.5 molecules) to GC polymers resulting in amphiphilic GC-CA conjugates (Fig. 1a). The amphiphilic GC-CA conjugates were self-assembled into CNPs under aqueous conditions, induced by hydrophobic interactions between hydrophobic CA moieties conjugated to GC polymers. Finally, DOX-CNPs were prepared by the simple dialysis method to encapsulate hydrophobic DOX under DMSO/water co-solvent conditions^23^,^24^,^25^ (Fig. 1b). As CNPs possess many hydrophobic CA hydrophobic inner cores, the nanoparticles are stable in aqueous solution and also encapsulate hydrophobic DOX within the core. Second, thiolated GCs (tGCs) were also synthesized to utilize as a siRNA delivery vehicle, as previously reported^18^,^26^ (Fig. 1c). Briefly, in order to generate stabilized and condensed siRNA-CNPs, tGCs were synthesized by conjugation of sulfo-LC-SPDP to the primary amine of the GCs. Under optimal conditions, 7 mol% of amine residues of GC polymers were chemically modified into thiol groups and used to fabricate siRNA-CNPs. Also, Bcl-2 targeted polymerized siRNA (Poly-siRNA) was prepared through self-polymerization of each thiol group at the 5′ end of the sense and anti-sense strand of siRNA. Poly-siRNA and tGC were allowed to form a nanoparticle structure by self-assembly, wherein weak charge interactions between the positively charged tGC polymer and negatively charged Poly-siRNA initiated a process that was further stabilized by disulfide crosslinking between thiol groups of tGC polymers and Poly-siRNA in a mild buffer (Fig. 1d). The resulting siRNA-CNPs can be introduced into the cytosol of targeted cancer cells and then free siRNA can be released from CNPs under reductive conditions present within the cytosol^9^,^10^. Finally, free siRNA may lead to target-specific gene silencing through an RNAi mechanism in targeted cells.

Consequently, both CNP-based delivery vehicles will exhibit similar physicochemical properties such as size, surface properties and pH sensitive behavior, even after encapsulation of drugs with substantially different physical features. That is because the outermost surfaces of both DOX-CNPs and siRNA-CNPs are covered by the same GC polymers. Notably, that aspect apparently has resulted in the higher tumor accumulation and localization tendencies of CNP-based drug carriers by the enhanced permeability and retention (EPR) effect at tumor tissues^24^,^25^. Thereafter a similar degree of cellular uptake is anticipated, followed by intracellular trafficking to the site of action^18^, i.e. cytosol for RNAi processes and nucleus for DOX action, which will provide a combinatorial anti-cancer effect (Fig. 1e).

**Figure 2:**
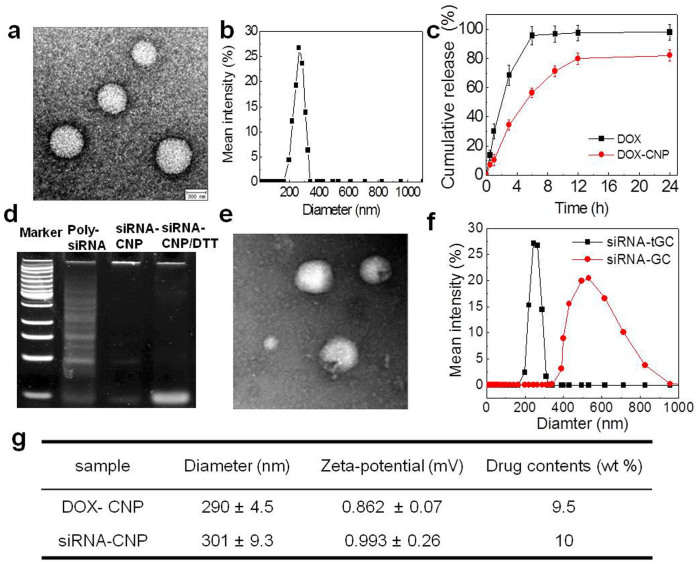
Physico-chemical properties of GC-based nanoparticles. (a) TEM images, (b) hydrodynamic size distribution and (c) *in vitro* DOX release of DOX-CNPs. When DOX was encapsulated into CNPs, the DOX release was effectively retarded even in the presence of 0.1% Tween 80 compared to the free DOX. (d) In polyacrylamide gel electrophoresis (PAGE) gel, the optimal weight ratio of Poly-siRNA to tGC polymers to produce condensed nanoparticles was determined as 1:10. Importantly, the condensed siRNA-CNPs were successfully dissociated into monomeric double-stranded siRNA after incubation with 10 mM dithiothreitol (DTT). (e) TEM images and (f) hydrodynamic size distribution of CNPs where the GC polymers with and without thiol groups. The complexes of Poly-siRNA/GC polymers without thiol groups showed larger particle sizes of 580 ± 58.5 nm compared to those of Poly-siRNA/tGC, indicating that additional disulfide crosslinking is a very critical process to form stabilized and compact nanoparticle. (g) Both GC-based nanoparticles exhibit almost the same physical properties such as diameter, zeta-potential and drug content even after encapsulation of siRNA and DOX that exhibit quite different physical properties.

The physicochemical properties of both nanoparticles were investigated in order to explain their *in vivo* behaviors. First, TEM image and dynamic light scattering (DLS) data showed that DOX-CNPs formed spherical nanoparticles with a mean diameter of 290 ± 4.5 nm in a PBS solution (Fig. 2a and 2b). The surface charge of DOX-CNPs was nearly neutral under those conditions, at 0.86 mV. The drug loading efficiency of DOX-CNPs was 93% at 10 wt% feed ratio of DOX to CNPs. The DOX release was effectively retarded even in the presence of 0.1% Tween 80 when DOX was encapsulated to CNPs, compared to free DOX (Fig. 2c). Second, in the case of siRNA-CNPs, thiol-modified sense and anti-sense strands of siRNA were initially prepared by self-polymerization and annealing in mild oxidative conditions. The Poly-siRNA, with an average of 12 siRNAs, easily formed a stable nanoparticle with tGC polymers by weak charge-charge interactions and further disulfide crosslinking between them, compared to unmodified siRNA. In a gel retardation assay, the optimal weight ratio of Poly-siRNA to tGC polymers to produce condensed nanoparticles was 1:10 (Fig. 2d). Importantly, the condensed siRNA-CNPs were successfully dissociated into monomeric double-stranded siRNA after incubation with 10 mM dithiothreitol (DTT) for 30 min, indicating that siRNA-CNPs may freely release uncondensed siRNAs under reductive conditions, such as obtained in the cytosol, even after polymerization and complexation with tGCs. The average hydrodynamic diameter of the spherical siRNA-CNPs was also confirmed as about 301 ± 9.3 nm in the physiological solution by TEM and DLS measurements (Fig. 2e and 2f). As a control, the complexes of Poly-siRNA/GC polymers without thiol groups showed a larger particle size of 580 ± 58.5 nm compared to those of Poly-siRNA/tGC. That result strongly indicates that the disulfide crosslinking between Poly-siRNA and tGC is a critical process to form stabilized and compact nanoparticle structures. The surface charge of siRNA-CNPs was nearly neutral (0.993 mV), indicating that the surface of siRNA-CNPs were mainly covered with GC polymers. As expected, the physicochemical properties of siRNA-CNPs are similar to those of DOX-CNPs, as summarized in Fig. 2g.

**Figure 3:**
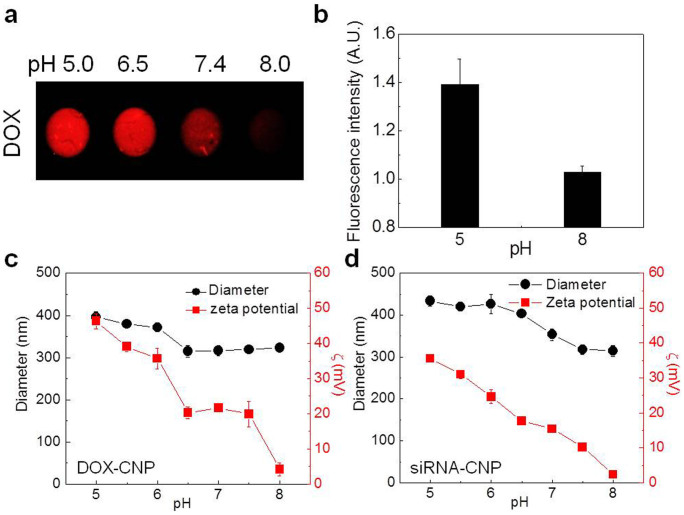
pH sensitive behaviors of both GC-based nanoparticles. (a) *In vitro* DOX release from DOX-CNPs with varying pH from 5 to 8 was monitored and (b) quantified by increased fluorescent signals. As a result, significant enhancement of DOX's fluorescent intensity was detected at acidic pH 5 compared to those of neutral and slightly alkaline pH. (c) Hydrodynamic sizes and (d) surface charges of DOX-CNPs and siRNA-CNPs were evaluated respectively over the pH range of 5–8. *In vitro* DOX release was significantly increased at acidic pH 5 compared to those of neutral and slightly alkaline pH. Furthermore hydrodynamic size and zeta-potential increased gradually with decreasing pH values.

**Figure 4:**
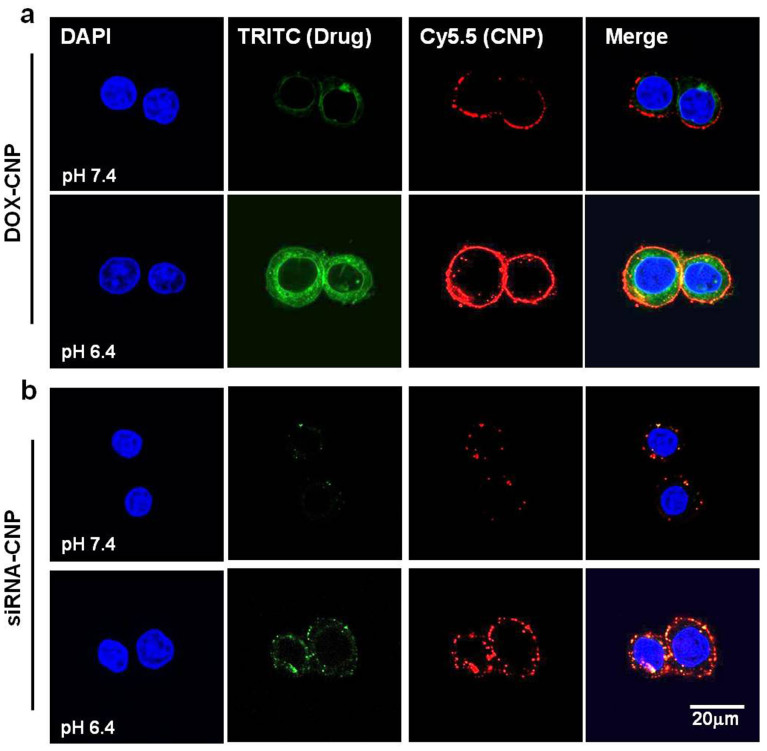
pH-dependent *in vitro* cellular uptake and intracellular trafficking of (a) DOX-CNPs and (b) siRNA-CNPs. GC polymers were labeled with Cy5.5 (Ex = 675 nm, Em = 695 nm, red color), and Poly-siRNA were labeled with TRITC (Ex = 557 nm, Em = 576 nm, green color). Both GC-based NPs were treated to PC-3 cells with pH 7.4 and 6.0 respectively and incubated for 3 h. Prominent cellular association of both fluorescent GC based NPs were observed only at weakly acidic pH 6.4 not at neutral pH.

The pH dependent behaviors of both DOX-CNPs and siRNA-CNPs were investigated *in vitro* to dissect the drug release profile and cellular uptake of both nanoparticles. When we measured DOX release from DOX-CNPs while varying pH from 5 to 8 by fluorescent imaging, prominent enhancement of the fluorescence intensity of DOX was detected at pH 5 relative to at the neutral and slightly alkaline pH values (Fig. 3a and 3b). That can be explained by the DOX release profile. Self-quenched encapsulated fluorescent DOX molecules at normal pH in the CNPs recover fluorescence after loosely bound DOX molecules are released at acidic pH. To verify the pH dependent DOX release behavior, hydrodynamic size and surface charge of DOX-CNPs and siRNA-CNPs were investigated, respectively over the pH range of 5–8 (Fig. 4c and 4d). With decreasing pH, hydrodynamic size and zeta-potential values of both CNPs increased gradually, and the zeta potential value of both CNPs approached zero near pH 8. When we consider that the pKa value of amino groups in the GC polymer is approximately 6.5^27^, the protonated amine groups of GC polymers under acidic conditions can ionize the GC polymers; consequently, CNP nanostructures can dissociate to release DOX and Poly-siRNA from the nanoparticle structure.

The *in vitro* cellular uptake experiment was used to explain the pH dependant behavior of both CNPs that may enhance the tumor cell-specific uptake of both nanoparticles. GC polymer was labeled with the near-infrared fluorescent dye, Cy5.5 (Ex = 675 nm, Em = 695 nm, red), and Poly-siRNA was labeled with TRITC (Ex = 557 nm, Em = 576 nm, green). When PC-3 cells were treated with both DOX-CNPs and siRNA-CNPs at pH 7.4 and 6.4 and incubated for 3 h, rapid cellular uptake of each CNP was only observed at weakly acidic pH 6.4 conditions and not at the neutral pH (Fig. 4a and 4b). Importantly, the large amount of DOX (green) and TRITC-Poly-siRNA (green) in both CNPs were clearly observed in the cytoplasm in acidified culture media compared to those at neutral pH (Fig. S1a and b). On the contrary, free DOX without CNPs were rapidly localized in the nucleus, and free TRITC-Poly-siRNA without CNPs did not enter the cells under either pH treatment condition (Fig. S1c). That is indicative of the different pharmacokinetic and intracellular behaviors of DOX and siRNA. From the cellular uptake data, we expect that the pH-dependent uptake of both CNPs can lead to strong electrostatic association with cancer cells, specifically in acidic tumor tissue *in vivo*^19^. At the normal pH of the blood stream and normal tissues, the surface charge of both CNPs is nearly neutral, which can prevent non-specific protein adsorption on the particle's surface^11^,^12^. Consequently, after reaching the target tumor site followed by effective pH dependent cellular association, both CNPs undergo different cellular trafficking, release behaviors of siRNA and DOX, and intracellular trafficking modes of CNPs and both drugs depending on the drug character and site of action.

Besides developing a well characterized delivery vehicle, a rational combination of chemotherapeutic agents, i.e. chemotherapeutics and genes, will be an important factor to enhance chemotherapy, especially by avoiding multi-drug resistance. In that context, a combination of Bcl-2 specific siRNA (siBcl2) and DOX has been a consistently attractive regimen. That is because Bcl-2 is an inhibitor of the mitochondrial apoptotic pathway and exerts its action by blocking pro-apoptotic counterparts Bad and Bax, thereby inhibiting the release of cytochrome C and activation of caspase-9^28^,^29^. To prove our claim, we choose a DOX/Bcl-2 siRNA combination since that combination was worked well in various tumor models, as well as was established well in various earlier studies. This is becaue DOX (apoptosis-induced anticacer drug) and Bcl-2 siRNA (suppressor of cellular anti-apoptotic defense mRNA) should give an effective efficacy in cancer therapy, when both drugs are successfully delivered to targeted cancer cell using new nanoparticle delivery system.

**Figure 5:**
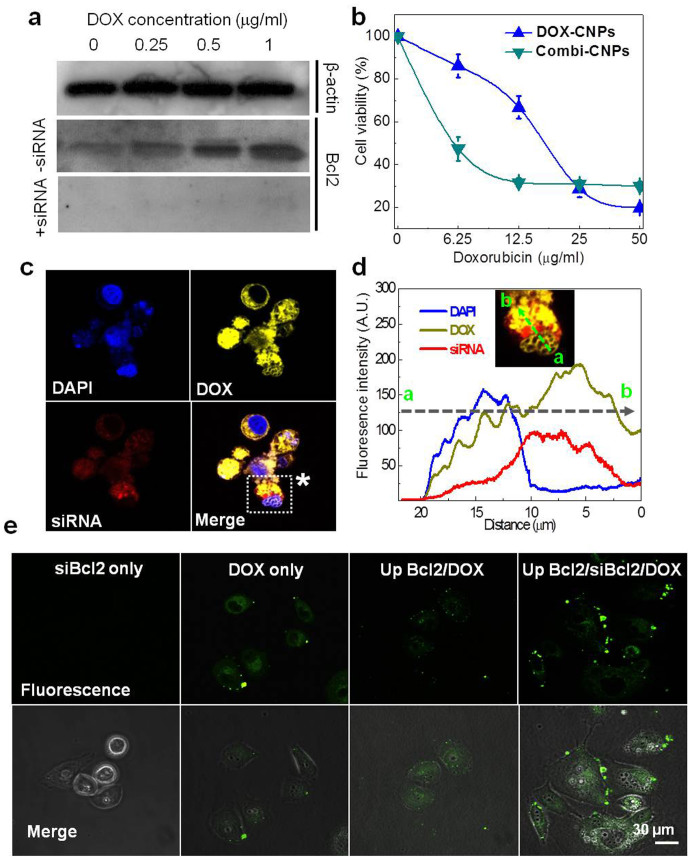
*In vitro* DOX and siBcl2 combinational effect on anti-cancer therapy. (a) Western blot analysis for the evaluation of DOX concentration and siBcl2 effect on Bcl-2 protein expression in drug resistance induced PC-3 cells. DOX-CNPs were treated on PC-3 cells for 2 h with varying DOX concentration from 0 to 1 μg/ml and further incubated for 24 h. Consequently the gradual increase of Bcl2 protein expression was detected prominently by western blot as the DOX concentration gradually increased. (b) CCK analysis was performed to evaluate the combinational effect of DOX-CNPs and siBcl2-CNPs after repetitive DOX-CNPs treatment to induce drug resistance. (c) Confocal microscopy images and (d) fluorescence intensities of DOX and siRNA in PC-3 cells after 24 h incubation. (e) Apoptosis detection by Annexin-V staining after the repetitive treatment of DOX-CNPs.

First, we evaluated whether repetitive treatment of DOX can induce multi-drug resistance by the cellular defense mechanisms and impede therapeutic effects. When PC-3 cells were treated with DOX-CNPs for 2 h at varying DOX concentrations (0 to 1 μg/mL) and further incubated for 24 h, the gradual increase of Bcl-2 protein expression was detected by western blot as the DOX concentration gradually increased (Fig. 5a). However, Bcl-2 protein bands noticeably disappeared after a single sequential treatment of siBcl2-CNPs, 1 day post-incubation of DOX-CNPs. Scrambled siRNA-CNPs did not show any Bcl-2 protein expression under the same conditions (Fig. S3). Additionally cell counting kit (CCK) analysis showed that the combinational treatment of DOX-CNPs and siBcl2-CNPs overcomes anti-apoptotic mechanisms effectively. In order to verify the effect of Bcl-2 expression on cell viability, drug resistant PC-3 cells were initially treated with three repetitive doses of DOX-CNP. Then DOX-CNPs or DOX-CNPs/siBcl2-CNPs (Combi-CNPs) were added to the drug resistant PC-3 cells, respectively, and cell viability was measured by CCK. In the Combi-CNPs group, the half maximal inhibitory concentration (IC50) value was 3.1-fold lower than that of the DOX-CNPs group (Fig. 5b), which relates to a more effective cell killing with a lower dose of DOX in the Combi-CNPs group. However, there was no effect on the cell viabilities when scrambles siRNA with DOX-CNP was treated with varying concentration compared to those of DOX-CNP (Fig. S4a). Also Cell viabilities test of scrambled siRNA-CNP, siBcl-2 only and siBcl2-CNP were confirmed after 2 days incubation (Fig. S4b). Those results suggest that Bcl-2 over-expression by DOX exposure is an important factor mediating drug resistance in PC-3 cells.

A combined effect of the two treatments is also suggested by the intracellular localization of both drugs and direct visualization of apoptosis induced by repetitive DOX exposure under the same conditions as the viability study. After treating PC-3 cells with both CNPs in 24 h, confocal microscopy revealed that DOX (yellow) and Cy5.5-Poly-siRNA (red) were localized in nucleus and cytosol, respectively, which are the sites of action for both drugs (Fig. 5c and 5d). That result indicates that the CNP platforms can deliver both drugs into cells and enable localization of the drug to its intracellular site of action. Additionally, confocal microscopy of annexin-V staining (green) showed that the repetitive dosing of DOX-CNPs suppressed the apoptosis of PC-3 cells (no green dots in PC-3 cells), while combined treatment with siBcl2-CNPs could overcome the anti-apoptosis mechanism as seen by the prominent green colored annexin-V, a marker of apoptosis present on the cellular membrane (Fig. 5e).

**Figure 6:**
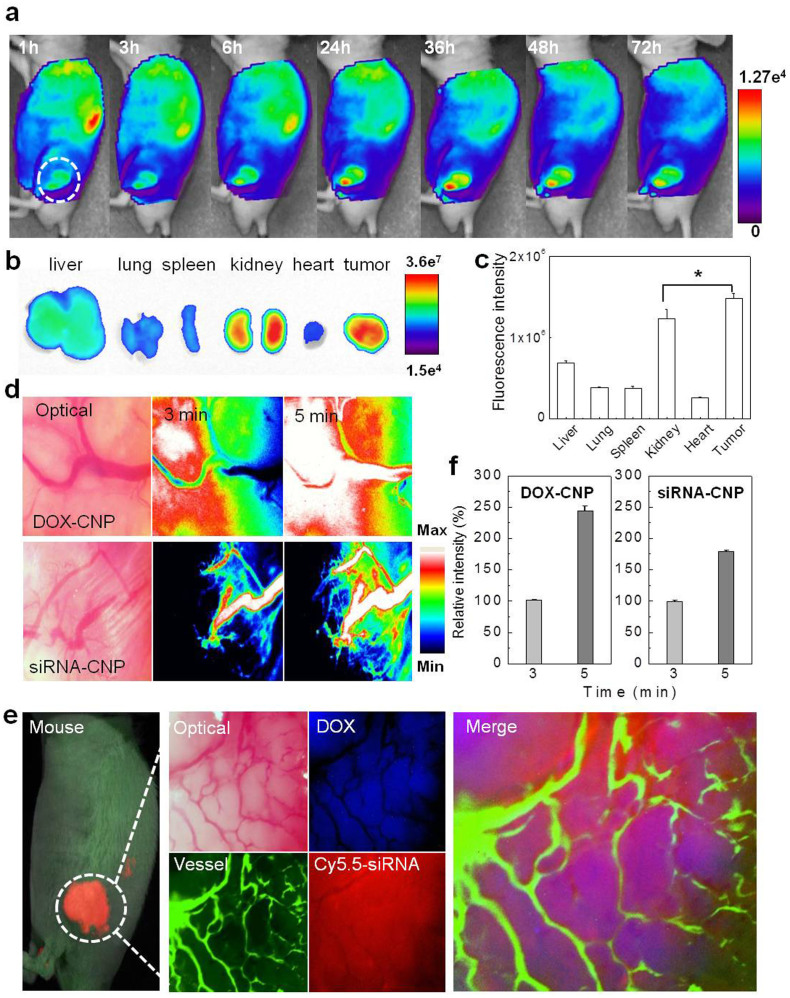
Evaluation of *in vivo* behaviors of both GC-based NPs by non-invasive near infrared fluorescence (NIRF) imaging system. (a) Time dependent whole body distribution of CNPs. (b) Fluorescence visualization and (c) quantification from the *ex vivo* NIR fluorescence images of dissected tumors and organs 72 h post-injection. (d,f) *In vivo* real-time tumor accumulation behaviors of both Cy5.5-CNPs in the solid tumor. (e) The multi-filtered live imaging of targeted release and localization of DOX and siRNA of CNPs in a solid tumor.

For optimal anti-tumor therapy, CNPs should possess (a) prolonged circulation in the body, (b) minimal nonspecific uptake in normal tissues, and (c) efficient targeting to solid tumors through EPR. Therefore, whole body distribution of DOX-CNPs and siRNA-CNPs were evaluated by near infrared fluorescence (NIRF) imaging^18^,^21^ in tumor-bearing mice. Cy5.5-DOX-CNPs (0.7 mg/kg of DOX, GC was labeled with 0.1wt% of Cy5.5) were injected intravenously into mice bearing PC-3 tumors, once the tumor was 200 ~ 250 mm^3^ in volume. Cy5.5-DOX-CNPs began to accumulate in the tumor tissue within 1 h post-injection, and subcutaneous tumor tissue could be delineated from the surrounding normal tissue, indicating the rapid tumor targeting ability of DOX-CNPs. The NIRF intensity of DOX-CNPs maximally increased at 24 h post-injection, and the tumors maintained this maximal NIRF intensity until 36 h post-injection. In addition, the NIRF intensity of DOX-CNPs was observed up to 72 h post-injection, indicating the long retention time of DOX-CNPs within tumor (Fig. 6a). Tissue distribution and tumor accumulation were precisely visualized and quantified from *ex vivo* NIRF of dissected tumors and organs, including liver, lung, kidney, spleen, and heart (Fig. 6b and 6c). The strongest NIRF intensities at 72 h post-injection were mainly observed in tumor. The total NIRF photon counts per gram of each organ was compared with the tumor tissue and was found to be 4–7 folds higher than in normal organs (liver, lung, spleen, and heart); whereas, strong NIRF intensity was also observed in the kidney, indicating renal clearance^19^,^30^. *In vivo* biodistribution and tumor targeting ability of siRNA-CNPs were very similar to that of DOX-CNPs (data not shown)^18^, indicating that the tumor targeting property of both DOX-CNPs and siRNA-CNPs were predominantly due to the physicochemical properties of the CNP-based delivery platform^19^.

*In vivo* real-time tumor accumulation of both Cy5.5-labeled CNPs containing DOX and siRNA was confirmed by the live imaging of blood vessels in tumor tissue. After intravenous injection of Cy5.5-labeled DOX-CNPs (0.7 mg/kg of DOX) and siRNA-CNPs (1.2 mg/kg of Poly-siRNA), NIRF signals indicating both CNPs were prominently observed inside the micro-vessels in the tumors within 3 min. Further, the fluorescent signals became stronger and diffused within deep tumor tissue as within 5 min (Fig. 6d and 6f). The fluorescent signals localized in the tumor tissue near the vessels suggests that both DOX-CNPs and siRNA-CNPs could extravasate from the vessel to the tumor effectively, indicating the decisive EPR effect of both nanoparticles. Furthermore, the targeted tumoral release and localization of both drugs in CNPs were also confirmed using multi-filtered live imaging (Fig. 6e). The fluorescence distribution of auto-fluorescent DOX (Ex = 480, Em = 560, blue) and Cy5.5-Poly-siRNA (Ex = 675, Em = 695, red) in DOX-CNPs and siRNA-CNPs were directly visualized after three daily IV injections of DOX-CNPs (0.7 mg/kg of DOX) for three days followed by a single siRNA-CNPs (1.2 mg/kg of Poly-siRNA) injection into PC-3 tumor-bearing mice. As expected, large fluorescent signals of both DOX and siRNA were distributed homogeneously in the tumor tissue near the blood vessels, confirmed by injecting dextran-FITC (Ex = 490, Em = 525, green), further prominent fluorescent signals in the tumors were observed in both CNPs (Fig. S5a). As a control, free DOX and Cy5.5-labeled Poly-siRNA were rapidly cleared from the body by 48 h post-injection (Fig. S5b), whereas DOX and siRNA in the CNPs accumulated and co-localized in the solid tumor together with both CNPs. The results suggest that encapsulation of different types of drugs into CNPs can overcome not only instability and poor biodistribution but also different *in vivo* behaviors such as pharmacokinetics and tendency for accumulation within tumor, consequently resulting in different therapeutic efficacies.

**Figure 7:**
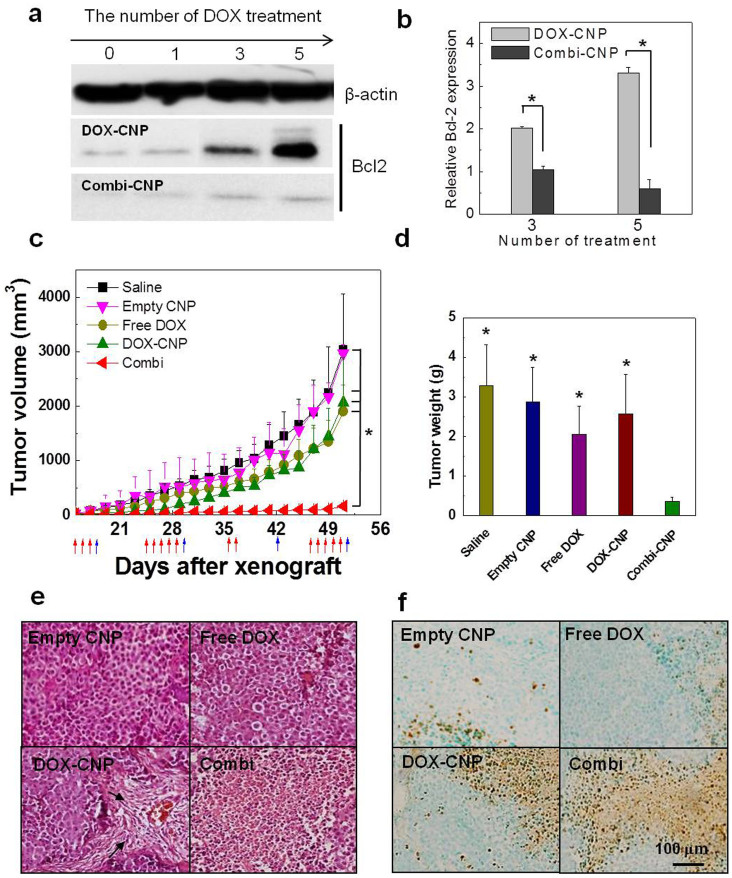
Comprehensive evaluation of *in vivo* therapeutic efficacies of combinational delivery. (a) Western blot bands and (b) quantification of Bcl-2 protein overexpression in PC-3 tumors after repetitive *i.v* injection of low concentrations of DOX-CNPs. (c) Tumor growth suppression efficacies and the (d) weight of excised tumor tissues 53 days post-treatment. (e) H&E and (f) TUNEL histological staining of the excised tumors.

To prove *in vivo* therapeutic efficacy of CNP-based sequential treatment, Bcl-2 protein over-expression of PC-3 tumor tissue was firstly induced after repetitive intravenous injections of a low concentration of DOX-CNPs (0.7 mg/kg of DOX). From western blot analysis, a single injection of DOX-CNPs did not present noticeable Bcl-2 protein expression 2 days post-injection, whereas repeated injections of DOX-CNPs 3 and 5 times per day significantly increased Bcl-2 expression (Fig. 7a). In order to confirm the effective inhibition of Bcl-2 overexpression by Bcl-2 gene knockdown, we injected the siRNA-CNPs (1.2 mg/kg of siBcl2) one day post-injection of DOX-CNPs. As a result, the sequential treatment of DOX and siBcl2 successfully decreased the expression level of Bcl-2 protein in the tumor tissue two days post-injection of siRNA-CNPs. Specifically a 2- and 5.6- fold decrease in Bcl-2 protein expression was confirmed after only a single injection of siBcl2-CNPs and 3 and 5 times of DOX-CNPs injections, respectively, indicating the specific gene silencing efficacy of systemically injected siRNA-CNPs in a tumor bearing mice model (Fig. 7b). Taken together, it is clear that the repetitive DOX-CNP injections per day stimulated Bcl-2 expression in solid tumor tissue, and the elevated Bcl-2 protein level was effectively suppressed by the sequential treatment of DOX-CNPs and siRNA-CNPs.

The therapeutic efficacy of the combination treatment of DOX-CNPs and siRNA-CNPs were evaluated in PC-3 tumor-bearing mice by comparing a single DOX-CNP treatment and sequential DOX-CNP and siRNA-CNP treatment (denoted as Combi-CNPs). When subcutaneous PC-3 tumors grew to 35 ± 5 mm^3^ in volume, comparative tumor growth inhibition efficacy studies were performed by dividing the animals into four groups (n = 6 per group); (*i*) saline, (ii) empty CNPs (7.4 mg/kg of CNPs) (*iii*) free DOX (0.7 mg/kg), (*iv*) DOX-CNPs (0.7 mg/kg of DOX), (*v*) DOX-CNPs (0.7 mg/kg of DOX) and siRNA-CNPs (1.2 mg/kg of siBcl2) (Combi-CNPs). Each group was intravenously administrated into PC-3 tumor bearing mice in a sequential way at predetermined times (red arrows: free DOX, empty CNPs or DOX-CNPs injection time; blue arrows: siRNA-CNPs injection time) (Fig. 7c). Prior to tumor growth inhibition test, we preliminarily confirmed that siBcl2-CNP alone did not effect on the tumor growth tendency compared to the Saline treated group (data not shown). And detailed description of the dose levels of each group was listed in the supplementary information (Table. S1). Also we determined the optimal interval of siRNA-DOX treatment as 10 day after preliminary test (data not shown). And low concentration of DOX-CNP was injected every day since repeated treatment of DOX was more effective in terms of therapeutic efficacy and inducing of drug resistance.

Therapeutic efficacy was examined by measuring tumor volumes over the course of 51 days. As a control, saline or empty CNP-treated mice did not present any therapeutic efficacy, and the tumor volumes gradually increased up to 51 days (tumor sizes were about 3,026 and 2,263 mm^3^, respectively). Importantly, only Combi-CNP-treated mice effectively suppressed the tumor growth rate up to 51 days, and the tumor size was 165 mm^3^ (mean ± s.e., n = 6, ANOVA at 95% confidence interval), significantly smaller than that of the free DOX and DOX-CNP-treated mice (1,900 mm^3^ and 2,063 mm^3^, respectively). However, free DOX and DOX-CNP-treated groups showed rapid tumor growth tendencies in the early stage of therapy. Interestingly, only the DOX-CNP-treated group showed sudden tumor growth 20 days post-treatment; moreover, the tumor growth rate became faster than that of the free DOX group and when the siRNA-CNPs treatment after 42 day post-injection of Combi-CNPs was quitted, sudden rapid growth of tumor volume was clearly observed (denoted as Combi (Q), Fig. S4). This result strongly indicates that effective delivery of DOX-CNPs to PC-3 solid tumors via excellent tumor homing efficiency may lead to more severe drug resistance to DOX treatment. Taking all these *in vivo* results into account, we conclude that the sequential treatment of DOX-CNPs and siRNA-CNPs could successfully overcome the chemo-drug resistance problem in cancer treatment. As expected, the weight of excised tumor tissues 51 days post-treatment were dramatically decreased up to 9.4 fold compared with saline, empty-CNPs, free DOX, and DOX-CNPs treated tumor tissues, respectively (Fig. 7d).

**Figure 8:**
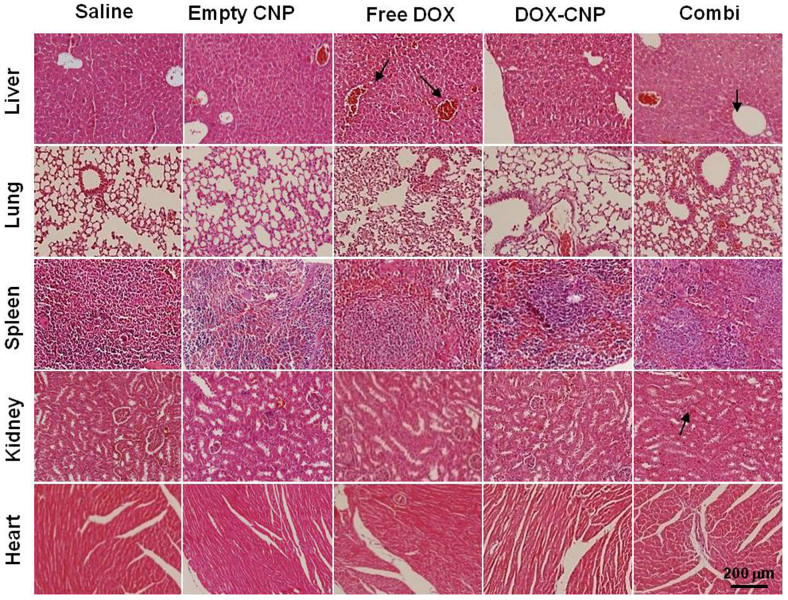
Evaluation of systemic toxicities by H&E staining showing histopathological changes in the major visceral organs.

We also performed H&E and TUNEL histological staining of the excised tumors (Fig. 7e and 7f). The PC-3 tumors of the control saline and empty-CNP-treated mice showed minimal cell death. Also, the free DOX treated group showed similar histological changes in tumors, indicating the severe drug resistant problem and inefficient tumor accumulation of free DOX. In contrast, tumors of the DOX-CNP-treated group included several small necrotic areas (black arrows) around the tumor vessels, due to the higher tumor targeting ability of DOX-CNPs. Combi-CNP-treated group presented a wide range of necrotic cell death across the broad tumor. In the TUNEL assay, the Combi-CNP-treated group showed distinct TUNEL positive areas surrounding broad necrotic areas of tumor cells compared to all other treatments. Finally, histopathological changes in the major visceral organs were also observed to evaluate systemic toxicity of each treatment (Fig. 8). Free-DOX-treated mice showed mild congestion in the liver (black arrows), but no distinct pathological lesions were discernable in other vital organs. Finally, DOX-CNP and Combi-CNP-treated mice did not show any pathological changes in the histology, except broad hemorrhaging in the spleen.

In this study, glycol chitosan (GC)-based nanoparticles (CNPs) were utilized as a new combinational drug delivery carrier for DOX and siRNA to achieve maximal therapeutic efficacy with minimal toxicities by overcoming chemo-drug resistance. Indeed, both DOX-CNPs and siRNA-CNPs have convincingly outstanding characters as highly specialized anti-tumor therapeutic delivery cargos such as drug loading contents, tumor accumulation ability, desirable biodistribution and biocompatibility. Through a well-organized experimental design with properly controlled nanoparticles, encapsulation of different types of drugs into the same type of nanoparticles can overcome not only instability and poor biodistribution but also different *in vivo* behaviors such as pharmacokinetics and tumor accumulation tendency. Consequently, different therapeutic efficacies can result depending on the intrinsic physicochemical properties of various chemotherapeutic agents. Based on the superior features of GC-based nanoparticles, significantly long-term tumor growth inhibition was achieved. Moreover, with precisely designed control groups, we clearly demonstrated that in order to maximize therapeutic efficacy, a proper administration method of several drugs as well as the proper combination of drugs are critical factors in overcoming drug resistance in cancer treatment.

## Methods

### Materials

Glycol chitosan (MW = 2.5 × 10^5^ Da; degree of deacetylation = 82.7%), 5β-cholanic acid, *N*-hydroxysuccinimide (NHS), 1-ethyl-3-(3-dimethylaminopropyl)-carbodiimide hydrochloride (EDC), anhydrous dimethyl sulfoxide (DMSO), 1,4-dithiothreitol (DTT), FITC-dextran, triethylamine (TEA), doxorubicin hydrochloride (DOX·HCl) and Mammalian Cell Lysis Kit were purchased from Sigma-Aldrich (St. Louis, MO, USA). Sulfosuccinimidyl 6-(3′-(2-pyridyldithio) propioamido) hexanoate (Sulfo-LC-SPDP) was purchased from Thermo Fisher Scientific Inc. (Rockford, IL, USA). Polymerized Bcl-2 siRNA (sense strand: 5′-SH-GUG AUG AAG UAC AUC CAU UdTdT-3′ and anti-sense stand 5′-SH-AAU GGA UGU ACU UCA UCA CdTdT-3′) was purchased from Bioneer Corporation (Daejeon, Korea). Near infrared fluorescence (NIRF) dye Flamma™ (FPR-675) was purchased from Bioacts (Incheon, Korea). Annexin V-FITC Apoptosis Detection Kit was purchaced from BD Biosciences (California, USA). Mouse monoclonal Bcl-2 (C-2) antibody and horseradish peroxidase (HRP) conjugated goat anti-mouse immunoglobulin (IgG-HRP) were purchased from Santa Cruz Biotechnology, Inc. (Texas, USA). All other chemicals were purchased as reagent grade and used without further purification. All solutions were made up in RNase-free distilled water. All the apparatuses were autoclaved prior to use.

Human prostate cancer cell line (PC-3) was purchased from the American Type Culture Collection (ATCC, Rochkville, MD, USA). For cell culture, RPMI-1640, trypsin-EDTA and fetal bovine serum (FBS) were purchased from Welgene Inc (Daegu, Korea). For transfection of cells, OPTI-MEM media was purchased from Gibco® (Life Technologies Corporation, USA).

### Synthesis of chemically modified glycol chitosan nanoparticels (CNPs)

To prepare hydrophobically modified glycol chitosan nanoparticles, glycol chitosan was chemically modified with 5β-cholanic acid via amide bond formation as described in previous reports^19^. In brief, 500 mg of glycol chitosan (2 μmol) was dissolved in 125 ml of distilled water/methanol mixture. 150 mg of 5β-cholanic acid (416 μmol) was dissolved in 125 ml of methanol. Then the solution was mixed with 120 mg of EDC and 72 mg of NHS for 30 min to introduce carboxylic acid groups to amine groups of GC polymers. The reactant was added into 5β-cholanic acid solution dropwisely and vigorously stirred for 24 h at room temperature. Then carboxylic acids introduced GC polymers were purified by dialysis against methanol/distilled water mixture (1:0, 1:1, 0:1 v/v) for 3 days using a cellulose membrane (MWCO = 12–14,000 Da, Spectrum®, Rancho Dominquez, CA, USA). The resulting solution was lyophilized to give white powder, hGC.

Thiolated GC polymers were also synthesized to deliver polymerized siRNA as described previously^14^,^22^. In brief, 30 mg of GC was dissolved in 10 ml phosphate buffer (pH 7.4, 10 mM) and mixed with 6 mg of sulfo-LC-SPDP. The mixture was vigorously stirred for 12 h at 25°C. Then, 18 mg of DTT was slowly added into GC-LC-SPDP solution for 3 h with adjusted pH at 3.5. The solution was purified by dialysis against distilled water for 2 days and lyophilized to give white powder, tGC.

To observe distribution of DOX-CNPs and siRNA-CNPs *in vitro* and *in vivo*, GC was modified with fluorophore, Cy5.5 (λ_ex_ = 675 nm, λ_em_ = 720 nm), via amide bond formation. In brief, 22 mg of GC was dissolved in 8 ml of DMSO. And then, 200 μg of Cy5.5-NHS (170 nmol in DMSO) was slowly added into GC solution. The reaction mixture was stirred overnight at room temperature and dialyzed against distilled water using a cellulose membrane (MWCO 12–14 kDa) for 2 days. The blue solution was lyophilized to give Cy5.5-GC. Cy5.5 conjugated hGC and tGC were prepared using Cy5.5-GC as described above.

### Preparation of DOX-CNPs and siRNA-CNPs

First, doxorubicin (DOX) encapsulated CNPs (DOX-CNPs) were prepared by simple dialysis method. In brief, 100 mg of hGC were dissolved in 20 ml of anhydrous DMSO/distilled water (1:1 v/v) and mixed with 42.8 mg of DOX (77.8 μmol) containing 2 ml of same co-solvent which pretreated 21.7 μl of TEA (155.6 μmol, 2 equiv.). Then, the solution was sonicated three times for 1 min each using a probe type sonicator (VCX-750, Sonics & Materials, CT, USA) and followed by dialysis for 12 h against water using dialysis membrane (MWCO = 12–14 kDa). After filtering with 0.8 μm syringe filter to remove unloaded DOX, the solution was lyophilized to give a red powder, DOX-CNPs.

To prepare tGC-siRNA complexes (siRNA-CNPs), 1 mg of tGC was dispersed in 400 μl of HEPES buffer (10 mM HEPES, 1 mM EDTA, pH 8.0) and the solution was sonicated for 1 min. Then, Poly-siRNA containing HEPES buffer (0.5 mg/ml) was slowly added into tGC solution at a weight ratio of 10. The mixture was incubated at 37°C for 1 h. The stable complex formation of siRNA-CNPs was confirmed by gel retardation assay before use.

### Characterizations of DOX-CNPs and siRNA-CNPs

The amount of encapsulated DOX in CNPs was determined using the UV-vis spectrometer (G1103A, Agilent, USA) by measuring absorbance at 490 nm (λ_ = max_ of DOX). In brief, 1 mg of DOX-CNPs was dissolved in DMSO/distilled water (1 ml, 1:1 v/v) and was analyzed based on a standard curve of free DOX. *In vitro* DOX release profile from DOX-CNPs was performed in 37°C PBS containing 0.1% of Tween 80. In brief, 8 mg of DOX-CNPs (760 μg of DOX) and free DOX (760 μg) were dispersed in 4 ml of PBS (pH 7.4). 1 ml of each solution was transferred into the dialysis membrane (MW cut off = 100 kDa) and gently shaken at 37°C in a water bath at 100 rpm. The medium was refreshed with 20 ml of fresh medium at the pre-determined time intervals. The amount of released DOX was quantified using the UV-vis spectrometer by measuring absorbance at 490 nm.

The siRNA decomplexation from siRNA-CNPs was evaluated by a gel retardation assay using 8% polyacrylamide gel electophoresis. In brief, the free poly-siRNA, siRNA-CNPs and siRNA-CNPs were incubated with 10 mM of DTT at 37°C for 1 h, in which the weight ratio of siRNA (1 μg) to tGC (10 μg) in siRNA-CNPs was fixed at 1:10. The decomplexated siRNA was visualized by electrophoresis, as described above.

The morphologies were observed using transmission electron microscopy (TEM, Philips CM30) at an accelerating voltage of 200 eKV. For TEM images, all samples were dispersed in distilled water and were stained by 2% uranyl acetate for negative staining. The drug loading content was calculated by a formula of drug weight ratio, i.e. drug weight (mg)/1 mg of CNP polymer + drugs (siRNA or DOX).

To observe pH dependent DOX release behaviors by detection of DOX auto-fluorescence recovery, 1 mg of DOX-CNPs was dispersed in PBS buffer for 30 min at 37°C at various pH (5.0–8.0). Then the fluorescence was measured using a KODAK Image Station 4000 MM (New Haven, CT, USA) using TRITC bandpath excitation/emission filter set (ex = 561 nm and em = 580 nm; Omega Optical).

To analyze pH dependent changes of size distribution and surface charge (ξ) of both GC-based nanoparticles, all samples were dispersed in PBS (pH 5.0–8.0, 500 μg/ml) and measured for size and zeta-potential using a Zeta-sizer (Nano ZS, Malvern, UK).

### *In vitro* Cellular uptake of DOX-CNPs and siRNA-CNPs

Observation of cellular uptake behavior of both CNPs were performed at pH of 7.4 and 6.0 respectively. In brief, PC3 cells (2 × 10^4^ cells) were seeded into 35 mm cover slide bottom dish and stabilized for 24 h. And then, PC3 cells were treated with pH adjusted (7.4 or 6.0) serum free RPMI1640 medium containing Cy5.5-DOX-CNPs (10 μg/ml of DOX) and serum free OPTI-MEM medium containing Cy5.5- siRNA-CNPs (100 nM of POPO™-3 intercalated siBcl2) for 3 h at 37°C. The cells were then washed twice with cold DPBS (pH 7.4) and fixed with 4% paraformaldehyde for 10 min. The nuclei were stained using DAPI for 10 min at room temperature. The cellular association and intracellular localization of GC-based nanoparticles were observed by confocal laser microscope (FV1000, Olympus, Japan). In addition to visualized intracellular localization of DOX and siRNA in cells, DOX-CNPs (10 μg/ml of DOX) and siRNA-CNPs (100 nM of Cy5.5- siBcl2) were treated for 24 h and observed by confocal microscopy. Fluorescence profile at asterisk(*) was analyzed using ZEN 2012 software (Carl Zeiss Microscopy GmbH, Germany).

### *In vitro* Bcl-2 protein expression by western blot analysis

To observe the effect of DOX-CNPs on *in vitro* Bcl-2 over-expression, 1 × 10^4^ of PC3 cells were seeded onto 6-well cell culture plates and maintained for 24 h for western blot analysis. Then, 0.1 μg/ml of DOX-CNPs (1 × 10^−6^ M DOX) was treated for 2 h and further incubated for 24 hr. Each well was washed 3 times with DPBS. The cells were then treated with siRNA-CNPs and scrambled siRNA-CNPs (100 nM siBcl2) for 24 h and washed 3 times with DPBS. For western blotting analysis, the cells were lysed using lysis buffer (Mammalian Cell Lysis Kit with 1% protease inhibitor, Sigma-Aldrich), and the lysates were centrifuged at 14,000 rpm for 20 min at 4°C. The total proteins of each sample was quantified by BCA assay (Pierce® BCA Protein Assay Kit, Thermo Scientific, USA) and 50 μg of proteins from each sample were mixed with sodium dodecyl sulfate (SDS) gel-loading buffer (125 mol/L Tris, pH 6.8, 5% glycerol, 2% SDS, 1% β-mercaptoethanol and 0.006% bromophenol blue) and boiled for 5 min. 30 μg of proteins were separated by 10% SDS-polyacrylamide gel electrophoresis and transferred onto nitrocellulose membrane (iBlot® Blotting System, Life Technologies Corporation). The membranes were blocked for 1 h at room temperature in 5% non-fat dried milk containing 1 × TBST solution (10 mol/L Tris, pH 7.4, 100 mol/L NaCl and 0.1% Tween 20). Then the membranes were incubated with 10 μg of mouse anti-human Bcl-2 antibody (C-2, Santa Cruz Biotechnology, Inc., USA) for 12 h at 4°C. The membranes were washed 3 times and incubated with 0.1 μg/ml of goat anti-mouse IgG-HRP antibody (Santa Cruz Biotechnology, Inc., USA) for 1 h. After 3 more washes, Bcl-2 protein band was detected with an ECL system.

### *In vitro* cytotoxicity test

PC-3 cells were cultured in RPMI 1640 medium containing 10% fetal bovine serum and 1% penicillin-streptomycin at 37°C in a CO_2_ incubator. The cytotoxic effect of DOX-CNPs and siRNA-CNPs in PC3 cells were estimated using a Cell Counting Kit (CCK) assay. In brief, 1 × 10^4^ of PC3 cells were seeded onto 48-well cell culture plates and maintained for 24 h. In order to verify the effect of Bcl-2 protein expression on cell viability by the repeated exposure of DOX treatment, firstly 12 μg/ml of DOX-CNPs (1 × 10^−6^ M DOX) was treated for 2 h, and each well was washed 3 times by Dulbecco's Phosphate Buffered Saline (DPBS) per day for 3 days. The cells were further treated with and without siRNA-CNPs (100 nM siBcl2) for 24 h to confirm inhibition of the anti-apoptotic pathway. Finally, various concentrations of DOX-CNPs (0 to 50 μg/ml of DOX) were treated for 24 h and replaced with 10% (v/v) CCK solution contained RPMI medium. After 1 h incubation, culture supernatant was analyzed at 450 nm using a micro-plate reader (VERSAmaxTM, Molecular Devices Corp., CA, USA).

### *In vitro* apoptosis detection by Annexin-V staining

To obtain high-resolution of apoptosis images at the cellular level, PC3 cells were seeded onto an 8-well chamber slide (5 × 10^3^ cells, Nunc® Lab-Tek® Chamber Slide System) and stabilized for 24 h. To up-regulate Bcl-2 protein, the cells were sequentially treated with DOX-CNPs and siRNA-CNPs with the same procedure of the *in vitro* cytotoxicity test. Apoptosis detection was performed using Annexin V-FITC Apoptosis Detection Kit and fluorescence of FITC exhibiting apoptosis phenomena was visualized using a confocal laser microscope (FV-10i, Olympus, Japan).

### *In vivo* fluorescent imaging (IVIS, OV-100)

In- vivo NIRF imaging was performed using the eXplore Optix system (Advanced Reasearch Technologies Inc., Montreal, Canada). Cy5.5-CNPs (8 mg/kg) was intravenously injected into PC3 tumor-bearing mice and fluorescence was monitored over time. For the NIRF imaging, laser power, exposure time and photon count time setting were optimized at 20 μW and 0.3 sec/point. NIRF emission was detected with a fast photomultiplier tube (Hamamatsu, Japan) and a time-correlated single photon counting system (Becker and Hickl GmbH, Germany). Tumor and major organs were dissected after 72 h post injection and monitored for their NIRF intensities using Advanced Molecular Imager X (Ami-X, Spectral Instruments Imaging, LLC., USA) at 5 sec exposure with medium binning. NIRF intensities of organs were quantified by Spectral Instruments Imaging Software (Spectral Instruments Imaging, LLC., USA).

Intravascular NIRF diffusion images in the live animal were obtained by using the Olympus OV-100 Whole Mouse Imaging System (Olympus Corp., Japan). In brief, PC3 tumor-bearing mice was anesthetized and an arc-shaped incision was made in the tumoral skin. After DOX-CNPs (8 mg/kg of Cy5.5-GC) and siRNA-CNPs (12 mg/kg of Cy5.5-GC) were administered via tail vein, the blood vessels on the outermost surface of the solid tumor and tumor tissues were observed over time. NIRF intensities were analyzed with Image-Pro Plus software 4.0 (Media Cybernetics, USA).

The intra-tumoral release and localization of both drugs, i.e. DOX and poly-siRNA, from the GC-based NPs was monitored IVIS Spectrum system (Xenogen Corporation, USA) and Olympus OV-100 Whole Mouse Imaging System. The fluorescence distribution of DOX and Cy5.5-siRNA in tumor tissue was visualized after the three daily *i.v.* injection of DOX-CNPs during 3 days followed by a single siRNA-CNPs injection. To distinguish between tumor vessel and tumor tissue, FITC-Dextran (8 mg/kg) was administered via tail vein before OV-100 imaging.

### *In vivo* anti-tumor therapeutic efficacy

All experiments with live animals were performed in compliance with the relevant laws and institutional guidelines of Korea Institute of Science and Technology (KIST), and institutional committees have approved the experiments (approved number of 2013-01-030). To demonstrate Bcl-2 expression behavior in an animal model, tumor-bearing athymic nude mice (5 weeks old, 20–25 g, male) were prepared by inoculating 1 × 10^6^ PC3 cells in RPMI 1640 medium (80 μl) into the left flank of mice. When tumors grew to approximately 100 ± 10 mm^3^ in volume, DOX-CNPs (0.7 mg/kg of DOX) were injected repeatedly 0, 1, 3 and 5 times respectively and siRNA-CNPs (1.2 mg/kg of siBcl2) were also injected into the mice via tail vein (n = 3 per each group). To evaluate the total amount of Bcl-2 protein in tumor tissues, all dissected tumor tissues were lysed using lysis buffer and analyzed by western blot as described above.

To observe therapeutic efficacy in the animal model, tumor-bearing athymic nude mice (5 weeks old, 20–25 g, male) were prepared by inoculating 1 × 10^6^ PC3 cells in RPMI 1640 medium (80 μl) into the left flank of mice. When tumors grew to approximately 35 ± 5 mm^3^ in volume, DOX-CNPs (0.7 mg/kg of DOX) and siRNA-CNPs (1.2 mg/kg of siBcl2) were sequentially injected into the mice via tail vein (n = 6 per each group). The therapeutic results of each group were evaluated by calculating the tumor volumes for 53 days and the total weight of tumor tissues were measured at 53 days. To determine toxicity, body weights, water and food amounts of each animal group were measured for 53 days.

### Histological analysis

For histological analysis, tumor tissue and organs were excised from mice at 53 days after inoculation, and they were fixed with 4% paraformaldehyde solution and embedded in paraffin. The sliced tumor tissue and organs (5 μm) were stained by hematoxylin and eosin (H&E) and observed by optical microscopy (BX 51, Olympus, USA). The TUNEL assay was performed using TdT-FragEL™ DNA Fragmentation Detection Kit. In brief, the tumor tissue slides (5 μm) were incubated with 20 μg/ml of proteinase K solution for 20 min at room temperature, and then they were washed with 1 × TBS. The slides were inactivated endogenous peroxidases and incubated with 1 × TdT equilibration buffer for 30 min. Then, TdT labeling reaction mixture was applied onto each slide and incubated at 37°C for 1.5 h. To terminate the labeling reaction, stop buffer covered the slides and was incubated for 5 min at room temperature. All tumor tissues were visualized using DAB solution and counterstained using methyl green solution.

### Statistics

The differences between experimental and control groups were analyzed using one-way ANOVA and considered significantly different (marked with an asterisk (*) in figures) at p < 0.05.

## Author Contributions

K.K. and S.S. designed the research, conducted the data analysis and co-wrote the manuscript. H.Y.Y. mainly performed all experiments. S.J.L., D.G.Y. and J.Y.R. performed the experiments. M.G.P., S.H.K. and I.C.K. provided overall guidance. J.H.P., M.S. and S.L. conducted the data analysis and assisted the experiment.

## Supplementary Material

Supplementary Information supplementary information

## References

[b1] Lam P. *et al.* The innovative evolution of cancer gene and cellular therapies. Cancer Gene Ther 20, 141–149 (2013). 23370333 10.1038/cgt.2012.93

[b2] Tredan O., Galmarini C. M., Patel K. & Tannock I. F. Drug resistance and the solid tumor microenvironment. J Natl Cancer Inst 99, 1441–1454 (2007). 17895480 10.1093/jnci/djm135

[b3] Danhier F., Feron O. & Preat V. To exploit the tumor microenvironment: Passive and active tumor targeting of nanocarriers for anti-cancer drug delivery. J Control Release 148, 135–146 (2010). 20797419 10.1016/j.jconrel.2010.08.027

[b4] Falci C., Morello E. & Droz J. P. Treatment of prostate cancer in unfit senior adult patients. Cancer Treat Rev 35, 522–527 (2009). 19487081 10.1016/j.ctrv.2009.04.014

[b5] Tomita Y. [Second-line treatment of advanced renal cell carcinoma after first-line targeted therapy]. Gan To Kagaku Ryoho 38, 907–910 (2011). 21677480

[b6] Sculier J. P. & Moro-Sibilot D. First- and second-line therapy for advanced nonsmall cell lung cancer. Eur Respir J 33, 915–930 (2009). 19336594 10.1183/09031936.00132008

[b7] Stahl M., Muller C., Koster W. & Wilke H. Second-line chemotherapy of advanced disseminated gastric cancer after cisplatin, infusional 5-fluorouracil, folinic acid (PLF): benefit dependent on progression-free interval after first-line therapy. Onkologie 28, 499–502 (2005). 16160395 10.1159/000087136

[b8] Shapira A., Livney Y. D., Broxterman H. J. & Assaraf Y. G. Nanomedicine for targeted cancer therapy: towards the overcoming of drug resistance. Drug Resist Updat 14, 150–163 (2011). 21330184 10.1016/j.drup.2011.01.003

[b9] Son S., Namgung R., Kim J., Singha K. & Kim W. J. Bioreducible Polymers for Gene Silencing and Delivery. Acc Chem Res 45, 1100–1112 (2012). 22129162 10.1021/ar200248u

[b10] Son S., Singha K. & Kim W. J. Bioreducible BPEI-SS-PEG-cNGR polymer as a tumor targeted nonviral gene carrier. Biomaterials 31, 6344–6354 (2010). 20537703 10.1016/j.biomaterials.2010.04.047

[b11] Son S. *et al.* RVG peptide tethered bioreducible polyethylenimine for gene delivery to brain. J Control Release 155, 18–25 (2011). 20800628 10.1016/j.jconrel.2010.08.011

[b12] Son S. & Kim W. J. Biodegradable nanoparticles modified by branched polyethylenimine for plasmid DNA delivery. Biomaterials 31, 133–143 (2010). 19783041 10.1016/j.biomaterials.2009.09.024

[b13] Rim H. P., Min K. H., Lee H. J., Jeong S. Y. & Lee S. C. pH-Tunable calcium phosphate covered mesoporous silica nanocontainers for intracellular controlled release of guest drugs. Angew Chem Int Ed Engl 50, 8853–8857 (2011). 21826770 10.1002/anie.201101536

[b14] Dong Y. *et al.* Lipid-like nanomaterials for simultaneous gene expression and silencing in vivo. Adv Healthc Mater 3, 1392–1397 (2014). 24623658 10.1002/adhm.201400054 PMC4160381

[b15] Jiang T. *et al.* Gel–Liposome-mediated co-delivery of anticancer membrane- associated proteins and small-molecule drugs for enhanced therapeutic efficacy. Adv Funct Mater 24, 2295–2304 (2014).

[b16] Chen A. M. *et al.* Co-delivery of Doxorubicin and Bcl-2 siRNA by Mesoporous Silica Nanoparticles Enhances the Efficacy of Chemotherapy in Multidrug-Resistant Cancer Cells. Small 5, 2673–2677 (2009). 19780069 10.1002/smll.200900621 PMC2833276

[b17] Tekedereli I. *et al.* Therapeutic Silencing of Bcl-2 by Systemically Administered siRNA Nanotherapeutics Inhibits Tumor Growth by Autophagy and Apoptosis and Enhances the Efficacy of Chemotherapy in Orthotopic Xenograft Models of ER (−) and ER (+) Breast Cancer. Mol Ther-Nucl Acids 2, e121 (2013). 10.1038/mtna.2013.45 PMC4028016 24022053

[b18] Lee S. J. *et al.* Tumor-Homing Poly-siRNA/Glycol Chitosan Self-Cross-Linked Nanoparticles for Systemic siRNA Delivery in Cancer Treatment. Angew Chem Int Ed Engl 51, 7203–7207 (2012). 22696263 10.1002/anie.201201390

[b19] Na J. H. *et al.* Real-time and non-invasive optical imaging of tumor-targeting glycol chitosan nanoparticles in various tumor models. Biomaterials 32, 5252–5261 (2011). 21513975 10.1016/j.biomaterials.2011.03.076

[b20] Rudzinski W. E. & Aminabhavi T. M. Chitosan as a carrier for targeted delivery of small interfering RNA. Int J Pharm 399, 1–11 (2010). 20732398 10.1016/j.ijpharm.2010.08.022

[b21] Kim K. *et al.* Tumor-homing multifunctional nanoparticles for cancer theragnosis: Simultaneous diagnosis, drug delivery, and therapeutic monitoring. J Control Release 146, 219–227 (2010). 20403397 10.1016/j.jconrel.2010.04.004

[b22] Chen M.-C. *et al.* The characteristics, biodistribution and bioavailability of a chitosan-based nanoparticulate system for the oral delivery of heparin. Biomaterials 30, 6629–6637 (2009). 19767097 10.1016/j.biomaterials.2009.08.030

[b23] Kim J. H. *et al.* Antitumor efficacy of cisplatin-loaded glycol chitosan nanoparticles in tumor-bearing mice. J Control Release 127, 41–49 (2008). 18234388 10.1016/j.jconrel.2007.12.014

[b24] Lee S. J. *et al.* Tumor specificity and therapeutic efficacy of photosensitizer-encapsulated glycol chitosan-based nanoparticles in tumor-bearing mice. Biomaterials 30, 2929–2939 (2009). 19254811 10.1016/j.biomaterials.2009.01.058

[b25] Lee S. J. *et al.* Comparative study of photosensitizer loaded and conjugated glycol chitosan nanoparticles for cancer therapy. J Control Release 152, 21–29 (2011). 21457740 10.1016/j.jconrel.2011.03.027

[b26] Mao S., Sun W. & Kissel T. Chitosan-based formulations for delivery of DNA and siRNA. Adv Drug Del Rev 62, 12–27 (2010). 10.1016/j.addr.2009.08.004 19796660

[b27] Riva R. *et al.* Chitosan and Chitosan Derivatives in Drug Delivery and Tissue Engineering. Adv Polym Sci 244, 19–44 (2011).

[b28] Shamas-Din A., Kale J., Leber B. & Andrews D. W. Mechanisms of action of Bcl-2 family proteins. Cold Spring Harb Perspect Biol 5, a008714 (2013). 23545417 10.1101/cshperspect.a008714 PMC3683897

[b29] Fernandez A. *et al.* Angiogenic potential of prostate carcinoma cells overexpressing bcl-2. J. Natl. Cancer Inst. 93, 208–213 (2001). 11158189 10.1093/jnci/93.3.208

[b30] Park K. *et al.* Effect of polymer molecular weight on the tumor targeting characteristics of self-assembled glycol chitosan nanoparticles. J Control Release 122, 305–314 (2007). 17643545 10.1016/j.jconrel.2007.04.009

