# Supplementary material for: Glycol chitosan nanoparticles as specialized cancer therapeutic vehicles: Sequential delivery of doxorubicin and Bcl-2 siRNA
Source: Sci Rep. 2014 Nov 3;4:6878. doi: 10.1038/srep06878 (PMC4217108; doi:10.1038/srep06878)
Supplement: Supplementary Information [file srep06878-s1.doc]

**Glycol chitosan nanoparticles as highly specialized cancer therapeutic vehicles: Sequential delivery of doxorubicin and Bcl-2 siRNA**

Hong Yeol Yoon,^†,‡,∥^ Sejin Son,^†,∥^ So Jin Lee,^†^ Dong Gil You,^†,‡^ Ji Young Rhee,^†^ Jae Hyung Park,^‡^ Maggie Swierczewska, Seulki Lee, Ick Chan Kwon,^†, §^ Sun Hwa Kim,^†^ Kwangmeyung Kim,^†^, *, Martin G. Promper,*


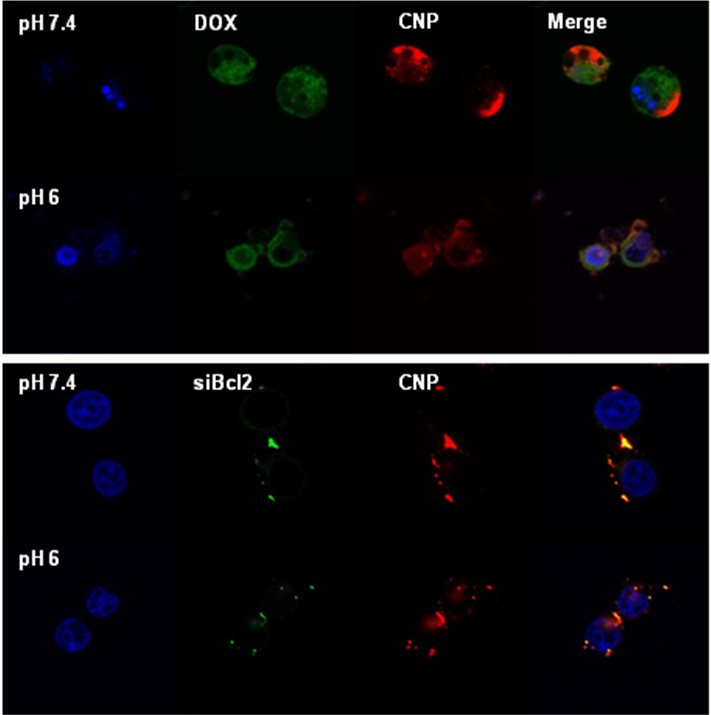


**Supplementary Figure 1a.** Intracellular uptake of DOX-CNP and siBcl2-CNPs at pH 6 and 7.4 after 48 hr post-incubation.


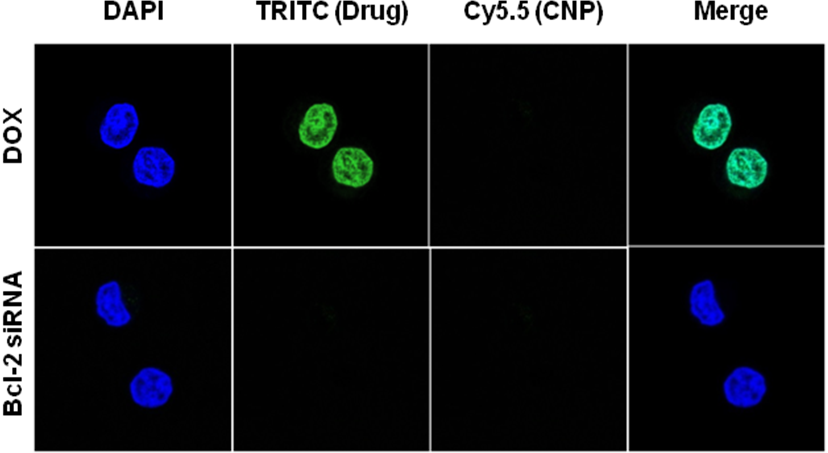


**Supplementary Figure 1b.** Intracellular uptake of free DOX and native siBcl-2 at 3 hr post-incubation. Free DOX without CNPs were rapidly localized in the nucleus, and free TRITC-Poly-siRNA without CNPs did not enter the cells at al.


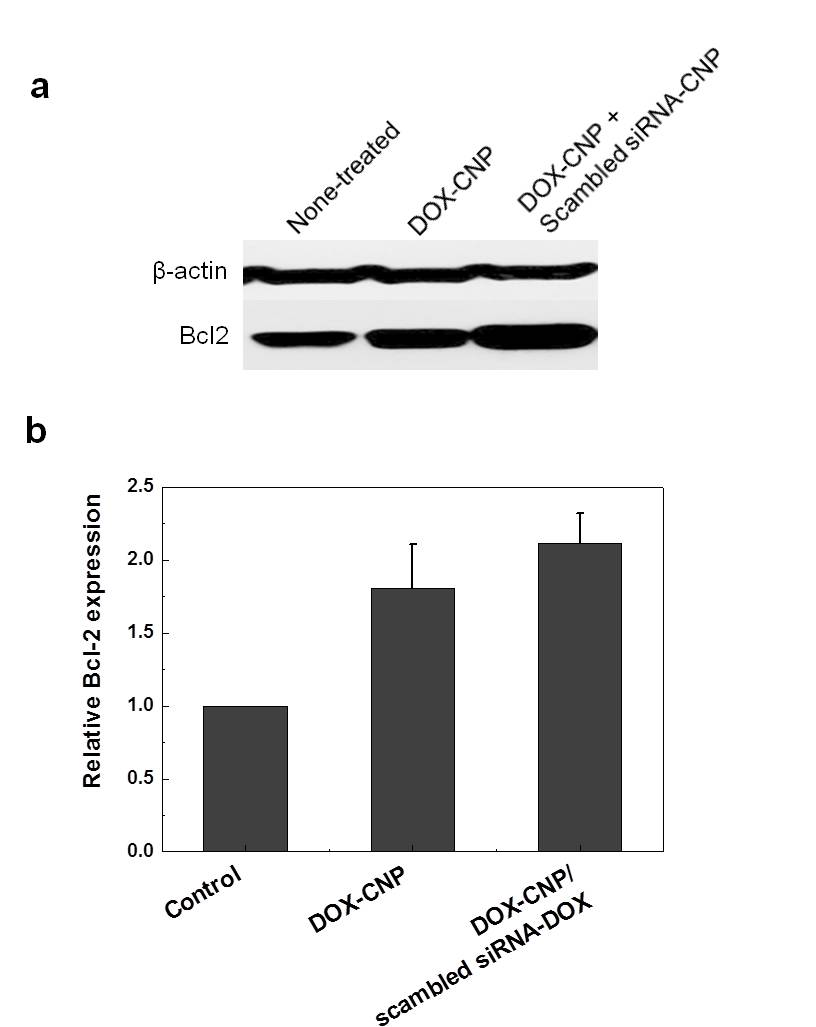


**Supplementary Figure 2.** Western blot analysis (a) and quantification (b) after treating DOX-CNP and DOX-CNP/scrambled siRNA-CNP. Both groups did not show any Bcl-2 protein expression in the same condition.


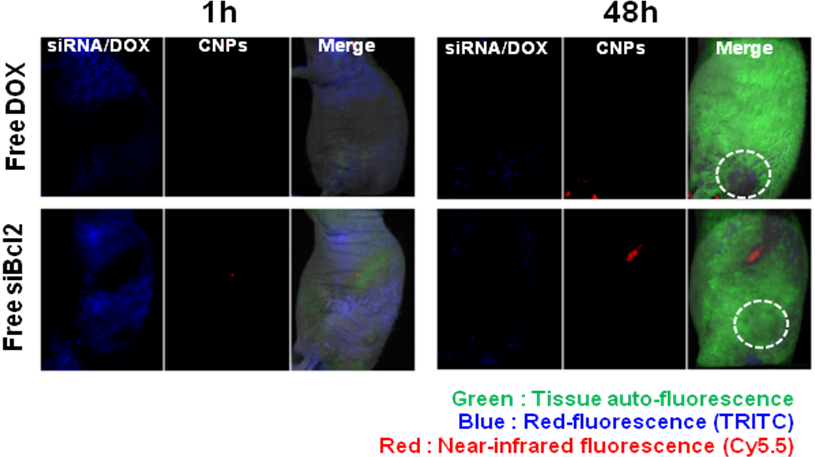


**Supplementary Figure 3.** The fluorescence distribution of auto-fluorescent DOX (Ex =480, Em=560, blue) and Cy5.5-Poly-siRNA (Ex =675, Em=695, red) were directly visualized after *i.v.* injections. As a result free DOX and Cy5.5-labeled Poly-siRNA were rapidly cleared from the body within 1 h post-injection.


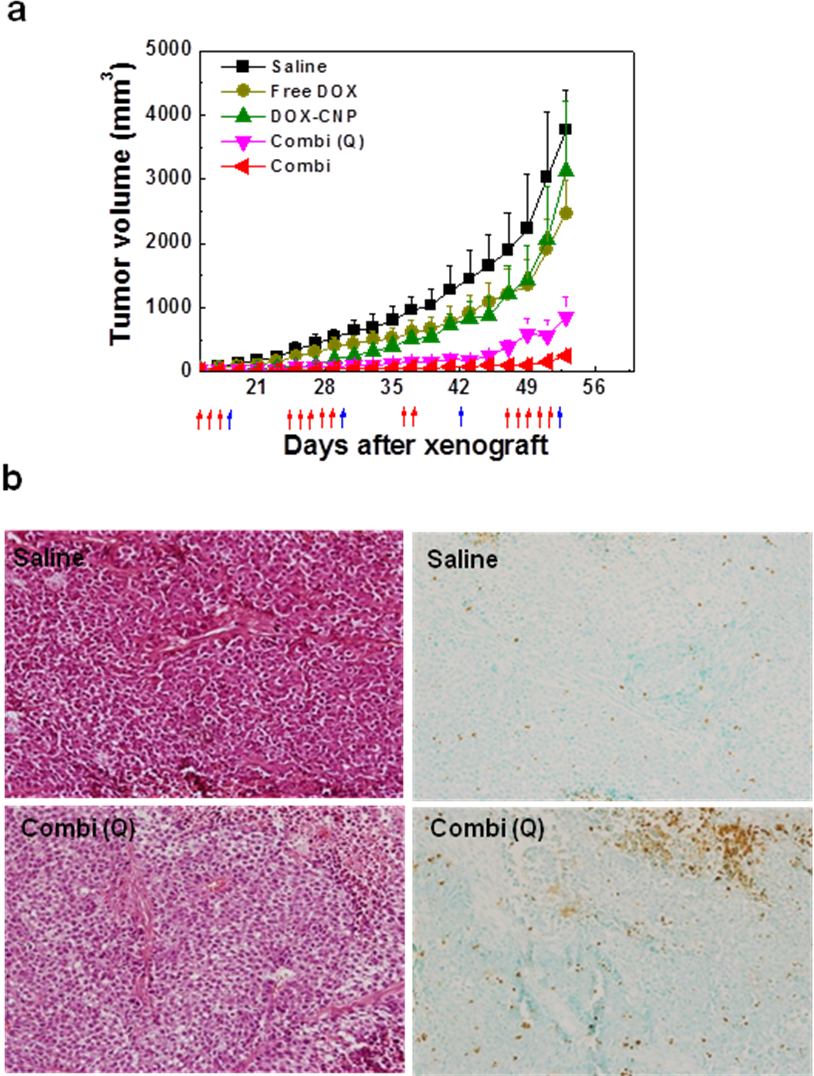


**Supplementary Figure 4.** Tumor growth inhibiton (a) and histological changes (b) of Combi (Q) group. When the dosing of siRNA-CNPs treatment after 42 day post-injection of Combi-CNPs was quitted, sudden rapid growth of tumor volume was clearly observed and cell death region was noticeably shrunk.


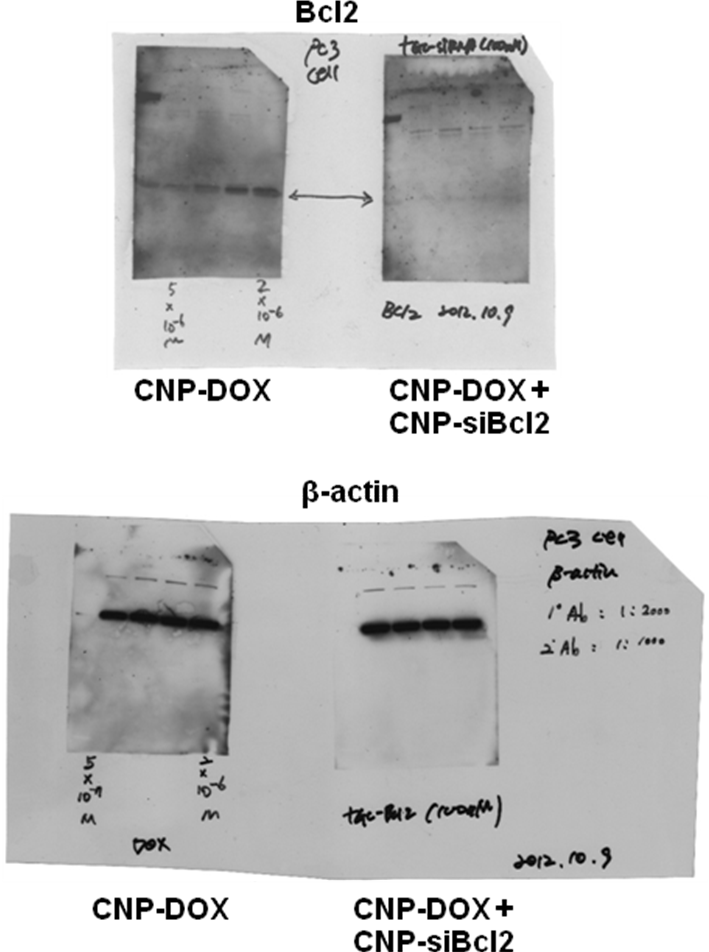


**Supplementary Figure 5.** Full length gel images for the western blot analysis of Figure 5a.

**
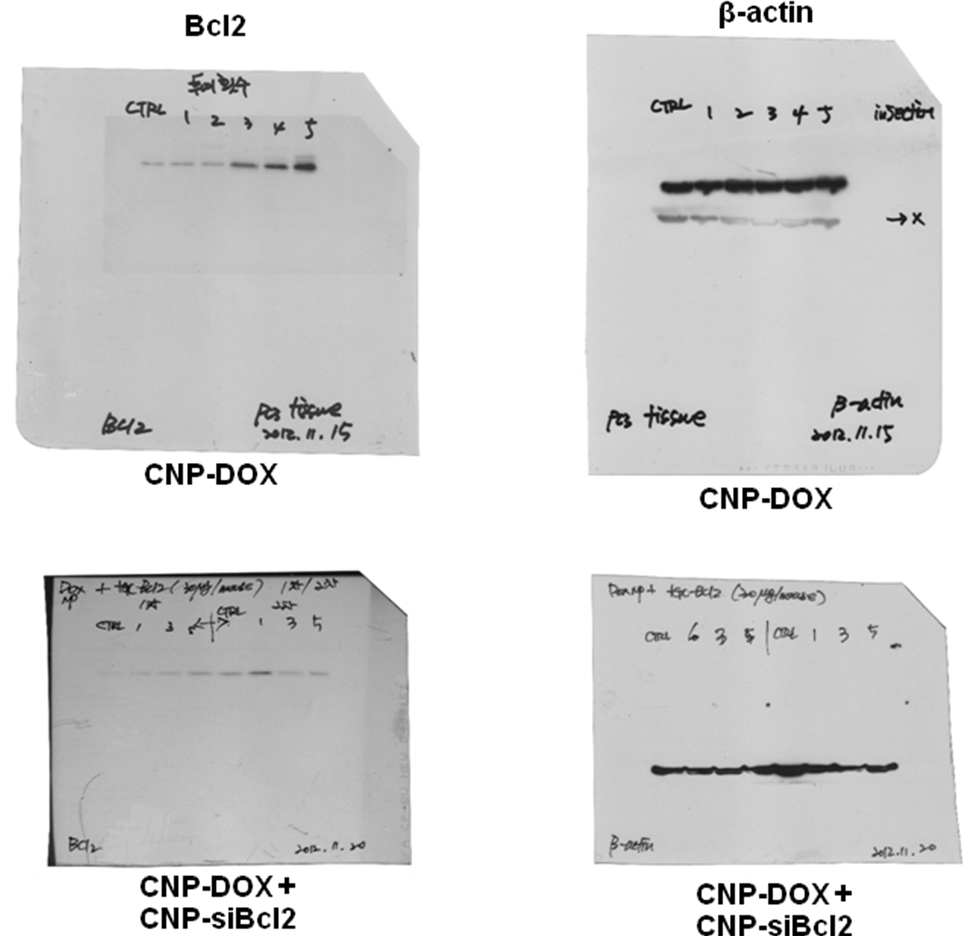
**

**Supplementary Figure 6.** Full length gel images for the western blot analysis of Figure 7a.


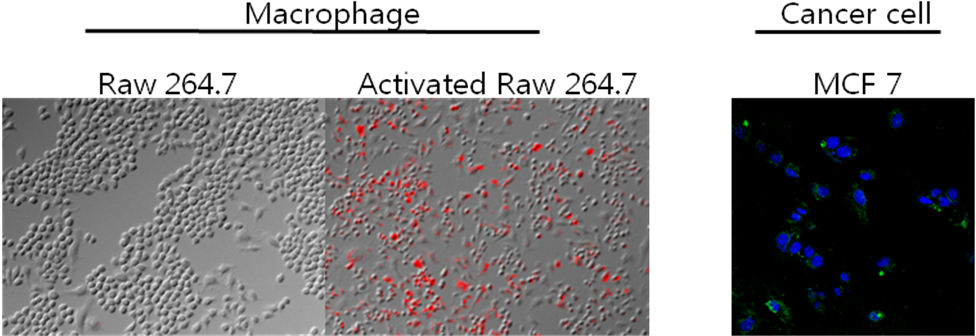


**Supplementary Figure 7.** Intracellular uptake tendency of CNP in various cell lines.

**Supplementary Figure 8.** Cytotoxicity and therapeutic efficacy tests of control groups Scrambled siRNA-CNP, siBcl-2 only and siBcl2-CNP were confirmed with or without DOX-CNPs after 2 days incubation. As a result, DOX-CNP showed slightly higher cell survival tendency compared to those of Free DOX. And co-treatment of scrambled siRNA with DOX-CNP did not affect the cell viability tendency of DOX- CNP only.


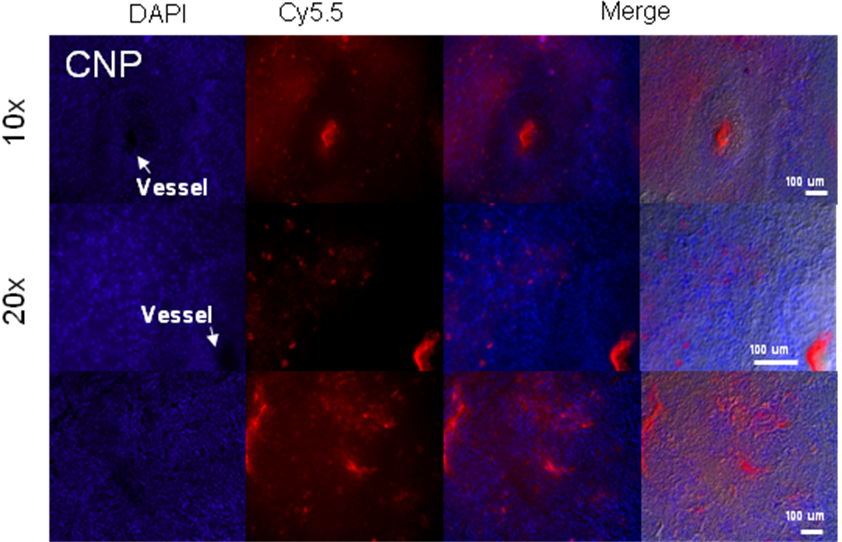


**Supplementary Figure 9.** CNPs accumulation into PC-3 solid tumor by histological section after tail vein i.v injection. As a result, prominent localization of fluorescent CNPs were observed inside the tumor vessels (white arrows). And around the vessels, red fluorescent CNPs were widely dispersed throughout the whole tumor tissues.
